# Commentary: Advances in Diagnosis and Management of Hemodynamic Instability in Neonatal Shock

**DOI:** 10.3389/fped.2018.00070

**Published:** 2018-03-26

**Authors:** David J. R. Hutchon

**Affiliations:** Obstetrics, Darlington Memorial Hospital, Darlington, United Kingdom

**Keywords:** vascular resistance, placenta, transition at birth, afterload of the heart, physiological transition

We congratulate Singh et al. on their article, which provides a comprehensive review of neonatal shock (1).

The neonatal myocardium has fewer contractile elements compared with older children and adults (2). In particular, immature myocardium has a higher basal contractile state and has higher sensitivity to changes in afterload (3). This is especially important in the context of the removal of placenta which is low vascular resistance state and transition to the higher vascular resistance state at birth.

However, this statement perpetuates the general misunderstanding about the vascular resistance of the placental circulation versus the systemic circulation. There is the implication that the vascular resistance of the placenta is unusually low. In fact, the vascular resistance of the healthy placenta is slightly *higher* than the vascular resistance of the systemic circulation. It is generally accepted that the placental circulation accommodates about 40% of the combined cardiac output (CCO) in the fetal circulation. Therefore, the systemic circulation accommodates most of the residual 60% with 10–15% going through the pulmonary circulation. In the parallel pattern of the fetal circulation, the same blood pressure drives both the placental and the systemic circulations, and with similar blood flows, the vascular resistances must also be similar.

As the authors point out, vascular resistance cannot be measured directly and can only be calculated from the measurements of blood pressure and flow, broadly following the same principles as OHMS law in an electrical circuit

$$\begin{array}{l} \text{ Resistance}=\text{pressure}/\text{flow }\\ \text{or Systemic Vascular Resistance}=\text{Blood Pressure}/\text{Cardiac Output.} \end{array}$$

The parallel circulation of the fetus. The total vascular resistance = blood pressure/flow, and the contribution of resistance by each of the circuits is calculated from the inverse of the total resistance.

1/R=1/r1+1/r2+1/r3.

In the fetal circulation, the placental, systemic, and pulmonary circulations are all in parallel and subject to the same blood pressure (Figure 1). Therefore, the vascular resistance of the systemic circulation, carrying about 50% of the CCO, must be slightly *lower* than the placental circulation carrying 40% of the CCO. Closure of *any* of these parallel circuits would result in an increase in the overall resistance. It is only the placental circulation that closes at birth, but the pulmonary circulation opens at the same time and by the end of transition accommodates about 50% of the CCO. Thus, in the neonate, the vascular resistance of the combined parallel circuits of the systemic circulation and the pulmonary circulation is similar to that of the three combined circulations in the fetal circulation. Separation of the systemic circulation and the pulmonary circulation with closure of the ductus arteriosus and foramen ovale divides the CCO equally between the left and right ventricles and into an adult pattern of serial circulation.

The higher vascular resistance state at birth described (1) is not therefore an inevitable part of a physiological transition.

**Figure 1:**
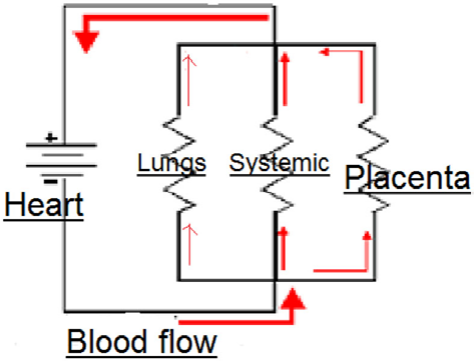
Electrical circuit equivalent of fetal circulation.

In fetal medicine, we measure placental blood flow with ultrasound Doppler to estimate the resistance index. A high resistance index is a sign of placental pathology.

It is true that the healthy placental circulation has a low vascular resistance, but it is approximately the same as the vascular resistance of the systemic circulation. A dramatic fall in the pulmonary vascular resistance, as demonstrated by the marked increase in pulmonary blood flow when the lungs fill with air at birth, provides an alternative low resistance parallel circuit as the placental circuit closes. Closure of the ductus arteriosus and foramen ovale converts the parallel circulation into two serial circuits. There is no theoretical need for an increased vascular resistance during transition and no theoretical need to put the immature myocardium at risk from increased afterload. After transition into two separate serial circulations, there is the ability of the pressure in the systemic circuit to rise to maintain cerebral circulation with the effect of gravity on the erect neonate while maintaining the low pressure in the pulmonary circuit with its thin walled alveolar capillaries (2).

## Author Contributions

The author confirms being the sole contributor of this work and approved it for publication.

## Conflict of Interest Statement

The author declares that the research was conducted in the absence of any commercial or financial relationships that could be construed as a potential conflict of interest.
